# Receptor Activity-modifying Protein-directed G Protein Signaling Specificity for the Calcitonin Gene-related Peptide Family of Receptors*[Fn FN2]

**DOI:** 10.1074/jbc.M116.751362

**Published:** 2016-08-26

**Authors:** Cathryn Weston, Ian Winfield, Matthew Harris, Rose Hodgson, Archna Shah, Simon J. Dowell, Juan Carlos Mobarec, David A. Woodlock, Christopher A. Reynolds, David R. Poyner, Harriet A. Watkins, Graham Ladds

**Affiliations:** From the ‡ Division of Biomedical Cell Biology, Warwick Medical School, University of Warwick, Coventry, CV4 7AL, United Kingdom,; the § Department of Pharmacology, University of Cambridge, Cambridge, CB2 1PD, United Kingdom,; the ¶ Department of Platform Technology and Science, GlaxoSmithkline, Hertfordshire, SG1 2NY, United Kingdom,; the ‖ School of Biological Sciences, University of Essex, Wivenhoe Park, Colchester, Essex, CO4 3SQ, United Kingdom,; the ** School of Life and Health Sciences, Aston University, Aston Triangle, Birmingham, B4 7ET, United Kingdom, and; the ‡‡ School of Biological Sciences and Maurice Wilkins Centre for Molecular Biodiscovery, University of Auckland, Auckland 1010, New Zealand

**Keywords:** G protein-coupled receptor (GPCR), molecular dynamics, molecular modeling, signal transduction, yeast, adrenomedullin, adrenomedullin 2, CGRP, RAMP, signal bias

## Abstract

The calcitonin gene-related peptide (CGRP) family of G protein-coupled receptors (GPCRs) is formed through the association of the calcitonin receptor-like receptor (CLR) and one of three receptor activity-modifying proteins (RAMPs). Binding of one of the three peptide ligands, CGRP, adrenomedullin (AM), and intermedin/adrenomedullin 2 (AM2), is well known to result in a Gα_s_-mediated increase in cAMP. Here we used modified yeast strains that couple receptor activation to cell growth, via chimeric yeast/Gα subunits, and HEK-293 cells to characterize the effect of different RAMP and ligand combinations on this pathway. We not only demonstrate functional couplings to both Gα_s_ and Gα_q_ but also identify a Gα_i_ component to CLR signaling in both yeast and HEK-293 cells, which is absent in HEK-293S cells. We show that the CGRP family of receptors displays both ligand- and RAMP-dependent signaling bias among the Gα_s_, Gα_i_, and Gα_q/11_ pathways. The results are discussed in the context of RAMP interactions probed through molecular modeling and molecular dynamics simulations of the RAMP-GPCR-G protein complexes. This study further highlights the importance of RAMPs to CLR pharmacology and to bias in general, as well as identifying the importance of choosing an appropriate model system for the study of GPCR pharmacology.

## Introduction

Calcitonin gene-related peptide (CGRP),^3^ adrenomedullin (AM), and adrenomedullin 2 (AM2, also known as intermedin) are members of the calcitonin peptide family (1). This family also includes calcitonin and amylin. CGRP, an extremely abundant neuropeptide, is widely distributed throughout the sensory nervous system. It is a very potent vasodilator released during neurogenic inflammation and is particularly implicated in the onset of migraine. It is also cardioprotective and is associated with both pro- and anti-inflammatory actions (2, 3). AM is produced by the vascular endothelium and has extensive effects on the cardiovascular system including stimulation of angiogenesis and the modulation of vascular tone (4–6). AM2 affects the vascular system in a similar manner to AM (7–9). Like CGRP, AM and AM2 are also cardioprotective, and their administration results in decreased blood pressure and increased speed of recovery from myocardial infarction (10, 11).

CGRP, AM, and AM2 activate three receptors that share a common class B G protein-coupled receptor (GPCR) subunit, the calcitonin receptor-like receptor (CLR) (12). In each receptor, CLR forms a heterodimer with receptor activity-modifying protein (RAMP) 1, 2, or 3. The formation of this heterodimer is obligatory for receptor function and efficient translocation of both subunits to the cell surface (13). Heterodimerization with RAMP1, RAMP2, or RAMP3 forms the CGRP, AM_1_, or AM_2_ receptor, respectively (13). The peptide ligands activate each receptor with differing potencies (1, 12).

Activation of all three CLR-based receptors by CGRP, AM, or AM2 generates increased cAMP production through coupling to the stimulatory G protein, Gα_s_ (1, 12, 14). However, CGRP, AM, and AM2 can signal through other pathways (1, 15, 16). Several studies have indicated that the CGRP family of receptors can also couple to Gα_i/o_ subunits, because their cAMP responses can be significantly increased through treatment with pertussis toxin (PTX), particularly in electrically excitable cells (17–20). The AM/AM_2_ receptor cAMP signaling in HEK-293 cells has also been shown to be PTX-sensitive (21). The existing information on the stimulation of signaling by CGRP, AM, or AM2 other than through the Gα_s_-cAMP pathway has been gained predominantly from physiological studies, and the relative signaling bias of CGRP, AM, and AM2 at the three CLR-based receptors, even for the cAMP pathway, remains to be determined.

The study of signaling bias *in vivo* is complicated by cross-talk from the wide range of signaling pathways present in certain cell lines or primary cell cultures. The *Saccharomyces cerevisiae* growth system (22) provides a robust assay that enables the examination of the coupling of a GPCR of choice to single G protein subunits. This is achieved through replacing the last five amino acids of the native yeast G protein with the corresponding sequence from the human G protein of choice (22, 23). This assay has recently been successfully employed to characterize the signaling pathways underlying glucagon-like peptide 1 (GLP-1) receptor response to GLP-1 and the many receptor agonist mimetics available (24, 25). Miret *et al.* (26) in 2002 very elegantly described the functional expression of the CLR with RAMP1 and RAMP2 in yeast. However, somewhat surprisingly, given the more recent interest in signaling bias, a further characterization of RAMP-CLR combinations in yeast has not been performed.

In this study we have utilized *S. cerevisiae* to express either RAMP1, -2, or -3 along with CLR to assess the coupling of the three CGRP family receptors to different human Gα subunits upon stimulation with CGRP, AM, or AM2. We demonstrate that all members of the CGRP receptor family successfully couple to GPA1/Gα_s_, GPA1/Gα_i_, and GPA1/Gα_q_ yeast chimeras and that the coupling preference of each receptor is dependent upon the stimulating ligand. The results obtained from the yeast system were verified in HEK-293 mammalian cell lines by the assessment of cAMP accumulation (which showed sensitivity to PTX) and mobilizations of intracellular calcium ((Ca^2+^)*_i_*). The data confirm that RAMPs alter the ability of each peptide to couple to G proteins; they also indicate that the G proteins influence the rank order of agonist potency at the different receptors. For CGRP, AM, and AM2 this means that potent activation of what would not generally be considered their “normal” receptors can be observed when alternative downstream pathways, such as stimulation of Gα_i_ or mobilizations of (Ca^2+^)*_i_*, are considered.

Considerable understanding of class B GPCR structure, function, and dynamics has been gained (27), primarily through molecular dynamics simulations (28–33). Consequently, to gain insight into the possible mechanisms behind our experimental results, we used molecular modeling and molecular dynamics simulations of RAMP complexes with CLR and the glucagon receptor (GCGR) to suggest a mechanism whereby the C-terminal tail of the RAMPs may influence G protein bias at the CLR. Finally we demonstrate that care is required when selecting an appropriate mammalian cell line to use when investigating G protein bias, as analysis of a HEK-293S cell line failed to show Gα_i_ coupling for any of the RAMP-CLR complexes, thus highlighting the fact that agonist bias can be directly influenced by the cellular background.

## Results

### 

#### 

##### Gα_s_ Coupling of CLR-based Receptors

**FIGURE 1.**
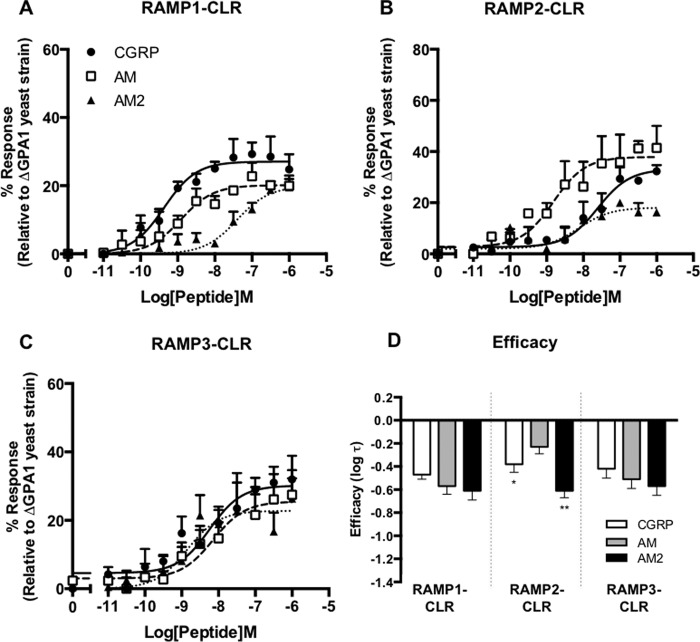
**Functional expression of CLR co-transformed with all three RAMPs in yeast cells.** Dose-response curves to CGRP, AM, and AM2 were constructed in yeast strains containing the GPA1/Gα_s_ chimera and expressing CLR with RAMP1 (*n* = 6) (*A*), RAMP2 (*n* = 7) (*B*), and RAMP3 (*n* = 8) (*C*). Reporter gene activity was determined following 20 h of stimulation with each ligand. Data are expressed as a percentage of the maximum response observed in yeast strain MMY11 (lacking GPA1) and are means ± S.E. of *n* individual data sets. *D*, *bar chart* showing the efficacy of each ligand for each RAMP-CLR combination as determined via application of the operational model of receptor agonism (see Ref. 34 and Table 1). Data were determined as statistically different from the cognate ligand for each receptor (*, *p* < 0.05; **, *p* < 0.01; ***, *p* < 0.001) using a one-way ANOVA with Bonferroni's post-test.

We co-expressed CLR under the control of the strong *PGK* promoter with RAMP1, RAMP2, or RAMP3 independently in a yeast strain containing a chimeric Gα subunit in which the C-terminal five amino acids of GPA1 had been replaced with those of mammalian Gα_s_, in order to study the coupling of the resultant receptors to a system expressing just a single G protein. Concentration-response curves were constructed for growth of *S. cerevisiae* for each RAMP-CLR combination (*i.e.* the CGRP, AM_1_, and AM_2_ receptors) using the agonists CGRP, AM, and AM2. When CLR was co-expressed with RAMP1, all three ligands appeared to generate an equivalent level of response but with differing potencies (Fig. 1*A* and Table 1). This generated a rank order of potency for the three ligands of CGRP > AM > AM2. Application of the operational model of pharmacological agonism (34) indicates that all three ligands exhibit similar efficacies (log τ) in yeast when CLR and RAMP1 are co-expressed (Fig. 1*D* and Table 1). RAMP2 co-expression with CLR generated a functional receptor (Fig. 1*B*) with rank ligand potencies of AM > AM2 = CGRP. AM2 appeared to behave as a partial agonist with a reduced log τ at the RAMP2-CLR heterodimer when compared with the other peptide agonists (Table 1). AM had a significantly higher efficacy (*p* < 0.05) than that displayed by CGRP. Expression of RAMP3 with CLR in *S. cerevisiae* generated a functional receptor where all three ligands activated GPA1/Gα_s_-coupled signaling with similar potencies and efficacies (Fig. 1*C*).

**TABLE 1:** **Summary of pharmacological parameters for various ligands upon expression of the CLR with each RAMP in yeast strains containing GPA1/Gα_s_, GPA1/Gα_i_, or the GPA1/Gα_q_ chimera** Data are the mean ± S.E. of *n* individual data sets. Statistical significance compared with the cognate ligand (*, *p* < 0.05; **, *p* < 0.01; ***, *p* < 0.001; ****, *p* < 0.0001) for each receptor heterodimer (CGRP for RAMP1 + CLR and AM for CLR with either RAMP2 or RAMP3) was determined by one-way ANOVA with Dunnett's post-test.

	RAMP1-CLR			RAMP2-CLR			RAMP3-CLR		
	CGRP	AM	AM2	CGRP	AM	AM2	CGRP	AM	AM2
**GPA1/Gα_s_**									
pEC_50_*^a^*	9.35 ± 0.2*	8.80 ± 0.4***	7.22 ± 0.3***	7.60 ± 0.3*	8.82 ± 0.3*	8.05 ± 0.3*	8.24 ± 0.2	8.15 ± 0.4	8.85 ± 0.3
*E*_max_*^b^*	27.10 ± 1.6*	20.39 ± 2.8***	20.65 ± 1.1***	30.34 ± 4.1	37.46 ± 3.5	19.90 ± 2.5***	30.17 ± 2.7	25.51 ± 3.6	22.80 ± 2.3
p*K_a_**^c^*	9.22 ± 0.2*	8.81 ± 0.3***	7.31 ± 0.3***	7.70 ± 0.3*	8.77 ± 0.3	8.10 ± 0.3	8.30 ± 0.3	8.10 ± 0.3	8.61 ± 0.4
log τ*^d^*	−0.43 ± 0.04	−0.59 ± 0.07*	−0.61 ± 0.08	−0.38 ± 0.08*	−0.23 ± 0.06	−0.61 ± 0.06**	−0.42 ± 0.08	−0.51 ± 0.08	−0.57 ± 0.08
*n*	6	6	6	7	7	7	8	8	8

**GPA1/Gα_i_**									
pEC_50_*^a^*	8.26 ± 0.5	8.38 ± 0.3 *	8.57 ± 0.2**	8.89 ± 0.2**	7.91 ± 0.2**	8.42 ± 0.5**	8.52 ± 0.2	7.89 ± 0.8	8.49 ± 0.2
*E*_max_*^b^*	19.80 ± 3.0*	34.20 ± 3.7***	41.5 ± 3.3****	24.43 ± 1.7*	24.49 ± 2.0*	15.71 ± 2.5*	22.60 ± 1.8*	26.71 ± 1.8*	15.71 ± 2.1*
p*K_a_**^c^*	8.40 ± 0.5	8.20 ± 0.3**	8.24 ± 0.2	8.64 ± 0.2	7.75 ± 0.2	8.30 ± 0.5	8.37 ± 0.2	8.00 ± 0.2	8.30 ± 0.3
log τ*^d^*	−0.70 ± 0.1**	−0.33 ± 0.07**	−0.18 ± 0.1***	−0.50 ± 0.04**	−0.51 ± 0.05*	−0.89 ± 0.1**	−0.56 ± 0.06*	−0.50 ± 0.05*	−0.78 ± 0.07*
*n*	6	6	6	6	6	6	7	7	7

**GPA1/Gα_q_**									
pEC_50_*^a^*	7.53 ± 0.1	7.26 ± 0.2	7.99 ± 0.2	7.14 ± 0.2	7.93 ± 0.2	9.22 ± 0.4*	6.19 ± 0.5*	7.83 ± 0.2	6.76 ± 0.25
*E*_max_*^b^*	26.50 ± 1.2	14.08 ± 1.2****	16.73 ± 1.1****	27.74 ± 2.3	29.03 ± 2.6	11.33 ± 1.3****	20.7 ± 4.2	25.56 ± 2.0	32.11 ± 3.7
p*K_a_**^c^*	27.40 ± 0.1	7.19 ± 0.2	7.91 ± 0.03	7.01 ± 0.2	7.78 ± 0.2	9.16 ± 0.4*	6.10 ± 0.6*	7.71 ± 0.2	6.60 ± 0.3
log τ*^d^*	−0.46 ± 0.03	−0.79 ± 0.04***	−0.70 ± 0.04***	−0.42 ± 0.05	−0.39 ± 0.05	−0.88 ± 0.08***	−0.63 ± 0.2	−0.48 ± 0.05	−0.34 ± 0.1
*n*	7	7	7	6	6	6	6	6	6

*^a^* The negative logarithm of the agonist concentration required to produce a half-maximal response.

*^b^* The maximal response to the ligand expressed as a percentage of that obtained from a yeast strain (MMY11) lacking GPA1.

*^c^* The negative logarithm of the equilibrium disassociation constant for each ligand generated through use of the operational model of agonism (34).

*^d^* Log τ is the coupling efficacy parameter of each ligand.

**FIGURE 2.**
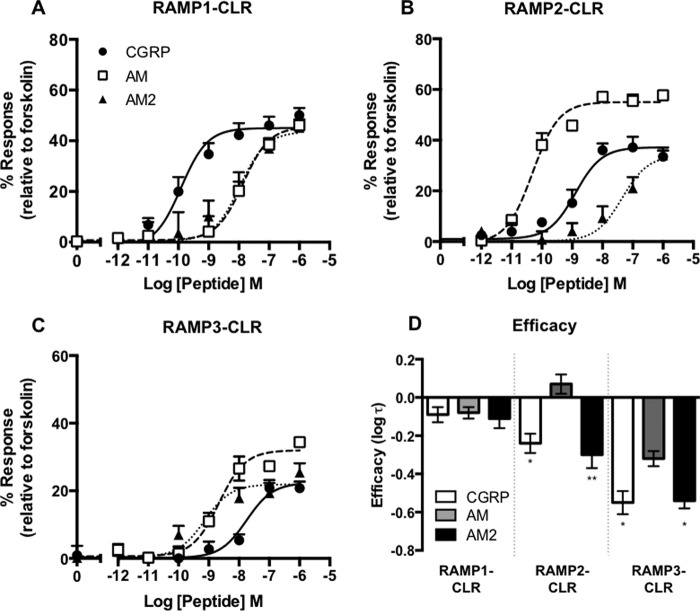
**Expression of CLR in combination with each RAMP generates functional Gα_s_-coupled receptors in HEK-293 cells.** cAMP accumulation was determined in HEK-293 cells transiently transfected with the CLR and RAMP1 (*n* = 11) (*A*), RAMP2 (*n* = 8) (*B*), and RAMP3 (*n* = 9) (*C*) following 30-min stimulation with CGRP, AM, and AM2. Data are expressed as percentage of cAMP production, determined using 100 μm forskolin stimulation, and are means ± S.E. of *n* individual data sets. *D*, *bar* chart showing the efficacy of each ligand for each RAMP-CLR combination as determined via application of the operational model of receptor agonism (34). Data were determined as statistically different from the cognate ligand for each receptor (*, *p* < 0.05; **, *p* < 0.01) using one-way ANOVA with Bonferroni's post-test.

We sought to confirm the pharmacology observed in the *S. cerevisiae* growth assay of the RAMP-CLR complexes in mammalian cell lines. For this we used HEK-293 cells that do not functionally express any RAMPs (25). Co-transfection of CLR and RAMP1 generated a rank order of ligand potency of CGRP ≫ AM = AM2. The rank order of ligand potency with co-transfection of CLR and RAMP2 was AM > CGRP ≫ AM2 and for CLR and RAMP3 was AM2 = AM > CGRP (Fig. 2 and Table 2). It is worth noting that, in our HEK-293 cells, only AM acted as a full agonist against the CLR when in complex with either RAMP2 or RAMP3. Overall the mammalian and yeast data showed similar results, with the most potent ligand at each receptor remaining the same in each case.

**TABLE 2:** **Potency (pEC_50_), affinity (p*K_a_*) and coupling efficacy (log τ) values for cAMP production at the CLR co-expressed with each RAMP and stimulated with various agonists measured in HEK-293 cells** Data are the mean ± S.E. of *n* individual data sets. Statistical significance compared with the cognate ligand (*, *p* < 0.05; **, *p* < 0.01; ***, *p* < 0.001) for each receptor heterodimer (CGRP for RAMP1-CLR and AM for CLR with either RAMP2 or RAMP3) was determined by one-way ANOVA with Dunnett's post-test.

	RAMP1-CLR			RAMP2-CLR			RAMP3-CLR		
	CGRP	AM	AM2	CGRP	AM	AM2	CGRP	AM	AM2
pEC_50_*^a^*	9.81 ± 0.20	7.92 ± 0.19**	7.93 ± 0.24**	8.97 ± 0.24***	10.35 ± 0.13	7.48 ± 0.23***	7.75 ± 0.3**	8.86 ± 0.14	9.14 ± 0.22**
*E*_max_*^b^*	45.0 ± 2.2	45.2 ± 3.7	43.6 ± 4.2**	37.2 ± 2.4***	55.0 ± 1.7	34.1 ± 4.0**	22.3 ± 2.1**	32.1 ± 1.6	21.9 ± 1.7**
p*K_a_**^c^*	9.60 ± 0.18	7.64 ± 0.28**	7.76 ± 0.20**	8.71 ± 0.2**	9.95 ± 0.23	7.16 ± 0.24**	7.64 ± 0.26**	8.50 ± 0.19	9.00 ± 0.18**
log τ*^d^*	−0.08 ± 0.04	−0.08 ± 0.09**	−0.11 ± 0.06**	−0.23 ± 0.05***	0.09 ± 0.05	−0.29 ± 0.07**	−0.54 ± 0.06**	−0.33 ± 0.04	−0.56 ± 0.04**
*n*	11	11	11	8	8	8	9	9	9

*^a^* The negative logarithm of the agonist concentration required to produce a half-maximal response.

*^b^* The maximal response to the ligand expressed as a percentage of the maximal cAMP production as determined using 100 μm forskolin stimulation.

*^c^* The negative logarithm of the equilibrium disassociation constant for each ligand generated through use of the operational model of agonism (34).

*^d^* Log τ is the coupling efficacy parameter of each ligand.

##### Gα_i_ Coupling of CLR-based Receptors

**FIGURE 3.**
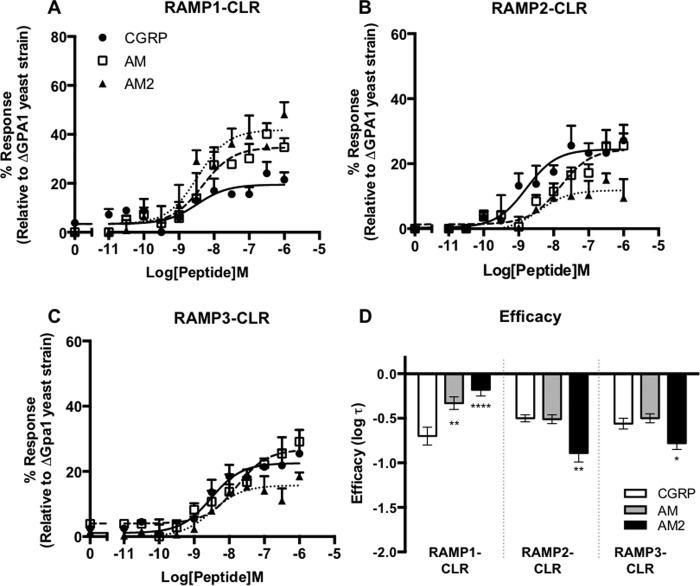
**Co-transformation of CLR with all three RAMPs in yeast cells generates receptors that couple functionally to the Gα_i_ chimera.** Dose-response curves to CGRP, AM, and AM2 were constructed in yeast strains containing the GPA1/Gα_i_ chimera and expressing CLR with RAMP1 (*n* = 6) (*A*), RAMP2 (*n* = 6) (*B*), and RAMP3 (*n* = 7) (*C*). Reporter gene activity was determined following 20 h of stimulation. All data are expressed as percentage of the maximum response observed in yeast strain MMY11 (lacking GPA1) and are means ± S.E. of *n* individual data sets. *D*, *bar* chart showing the efficacy of each ligand for each RAMP-CLR combination as determined via application of the operational model of receptor agonism (Ref. 34 and Table 1). Data were determined as statistically different from the cognate ligand for each receptor (*, *p* < 0.05; **, *p* < 0.01; ***, *p* < 0.001; ****, *p* < 0.0001) using a one-way ANOVA with Bonferroni's post-test.

To address the possibility that the CGRP family of receptors may couple not only to Gα_s_ but also to other subunits, we returned to the *S. cerevisiae* growth assay. In this case the yeast strain used contained a chimeric GPA1/Gα subunit including the last five residues of mammalian Gα_i_. We once again constructed concentration-response curves for yeast growth to the three agonists, CGRP, AM, and AM2. The co-expression of CLR and RAMP1 resulted in similar potencies for CGRP, AM, and AM2 (Table 1); however, AM and AM2 displayed significantly increased efficacy relative to CGRP for the activation of GPA1/Gα_i_ (Table 1 and Fig. 3, *A* and *D*). In contrast, when RAMP2 and CLR were co-transformed into the GPA1/Gα_i_ yeast strain, the rank order of ligand potency for GPA1/Gα_i_ yeast-based growth was CGRP > AM = AM2 (Table 1 and Fig. 3*B*). AM2 showed a significantly decreased efficacy compared with the other peptides (Table 1 and Fig. 3*D*). As with the RAMP1-CLR heterodimer, the combination of CLR and RAMP3 expressed in the GPA1/Gα_i_ strain resulted in similar potencies for CGRP, AM, and AM2 (Table 1 and Fig. 3*C*). However, AM2 displayed a significantly reduced efficacy when compared with AM (Table 1 and Fig. 3*D*).

In mammalian cells the Gα_s_ and Gα_i_ subunits act in opposition to regulate cAMP production. Therefore if a receptor can couple to both subunits in mammalian cells, the cAMP response measured is the result of a combination of the contribution from both pathways. Treatment of cells with PTX has been shown to uncouple receptors from the Gα_i_ subunit and therefore remove any inhibition of cAMP production. We sought to confirm the apparent Gα_s_-Gα_i_ coupling bias exhibited by the different RAMP-CLR combinations in the yeast reporter strains by measuring cAMP production from transiently transfected mammalian cells following PTX treatment.

**FIGURE 4.**
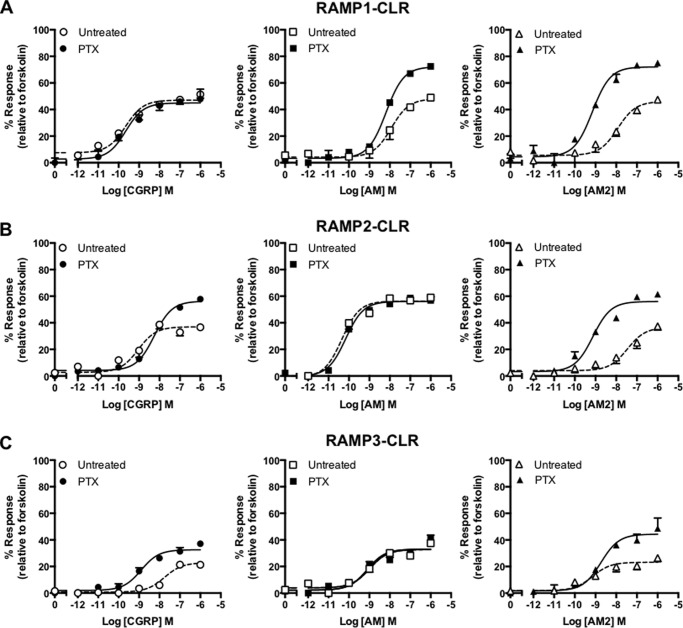
**CLR in combination with each RAMP generates receptors that display PTX-sensitive effects in response to ligand stimulation.** cAMP accumulation was determined in the presence (treated) and absence (untreated) of PTX from HEK-293 cells transiently transfected with CLR and RAMP1 (*n* = 6) (*A*), RAMP2 (*n* = 5) (*B*), and RAMP3 (*n* = 5) (*C*). Cells were stimulated for 30 min with CGRP, AM, and AM2. Data are expressed as percentage of the maximal cAMP production as determined using 100 μm forskolin stimulation in the presence of PTX and are means ± S.E. of *n* individual data sets.

Pretreatment of HEK-293 cells co-expressing RAMP1 with the CLR resulted in little overall increase in CGRP-mediated cAMP production (Fig. 4*A*). However, a significant elevation in *E*_max_ was observed in the same PTX-treated, RAMP1-CLR-expressing cells when challenged with either AM or AM2 (Fig. 4 and Table 3), suggesting that a Gα_i_ component for both of these ligands had been removed (Table 3). HEK-293 cells expressing CLR with either RAMP2 (Fig. 4*B*) or RAMP3 (Fig. 4*C*) displayed PTX-induced increases in *E*_max_ for cAMP accumulation following stimulation with both CGRP and AM2 (Table 3). However, for both combinations, the AM response appeared to be unaffected by PTX treatment, suggesting that little Gα_i_ coupling was present. Indeed, it is worth noting, that the cognate ligand for each receptor (CGRP for RAMP1-CLR and AM for RAMP2-CLR or RAMP3-CLR) did not appear to display an increased *E*_max_ upon PTX treatment, suggesting limited Gα_i_ components in these cases. Importantly, PTX treatment of untransfected HEK-293 cells did not result in a change in the overall levels of cAMP accumulation as determined by forskolin stimulation (untreated, 16.57 ± 2.5 pmol cell^−1^; treated, 16.45 ± 2.4 pmol cell^−1^), thereby confirming that the effects observed were specific to the RAMP-CLR combinations. Thus, there is abundant evidence that receptor and ligands can activate Gα_i_ in a mammalian cell, albeit in a complex pattern.

**TABLE 3:** **Potency (pEC_50_), affinity (p*K_a_*), and coupling efficacy (log τ) values for cAMP production at the CLR co-expressed with each RAMP, stimulated with various agonists measured in HEK-293 cells in the presence and absence of pertussis toxin** Data are the mean ± S.E. of *n* individual data sets. Statistical difference between PTX-treated and untreated cells was determined using Student's *t* test (*, *p* < 0.05; **, *p* < 0.01; ***, *p* < 0.001; ****, *p* < 0.0001).

	Untreated					Treated				
	pEC_50_*^a^*	*E*_max_*^b^*	p*K_a_**^c^*	log τ*^d^*	*n*	pEC_50_*^a^*	*E*_max_*^b^*	p*K_a_**^c^*	log τ*^d^*	*n*
**RAMP1**										
CGRP	9.66 ± 0.2	47.07 ± 2.2	9.43 ± 0.2	−0.11 ± 0.04	9	9.65 ± 0.2	44.95 ± 2.2	9.33 ± 0.3	−0.11 ± 0.07	6
AM	7.93 ± 0.2	48.06 ± 2.5	7.67 ± 0.2	−0.09 ± 0.05	9	8.14 ± 0.07	72.17 ± 1.7***	7.66 ± 0.2	− 0.36 ± 0.1**	6
AM2	7.93 ± 0.2	46.10 ± 4.1	7.70 ± 0.2	−0.11 ± 0.07	9	9.15 ± 0.1*0	72.15 ± 2.4***	8.56 ± 0.3	− 0.40 ± 0.1**	6

**RAMP2**										
CGRP	19.00 ± 0.2	36.97 ± 2.4	18.82 ± 0.2	−0.27 ± 0.05	9	18.25 ± 0.4	56.27 ± 1.4***	7.92 ± 0.2*	0 0.1 ± 0.06**	6
AM	10.35 ± 0.1	56.33 ± 1.6	10.00 ± 0.1	−0.07 ± 0.02	9	10.16 ± 0.07	56.07 ± 1.1	9.83 ± 0.2	0.07 ± 0.02	6
AM2	17.46 ± 0.2	36.61 ± 3.5	17.24 ± 0.2	−0.29 ± 0.07	9	19.13 ± 0.1**	56.05 ± 2.2***	8.84 ± 0.2**	00.1 ± 0.06*	6

**RAMP3**										
CGRP	7.75 ± 0.3	22.38 ± 2.6	7.64 ± 0.3	−0.54 ± 0.07	8	8.90 ± 0.1*	32.61 ± 1.5*	8.74 ± 0.2*	−0.29 ± 0.06	7
AM	8.98 ± 0.2	32.00 ± 1.5	8.83 ± 0.1	−0.33 ± 0.03	8	9.10 ± 0.2	35.95 ± 2.2	8.94 ± 0.2	−0.34 ± 0.05	7
AM2	9.10 ± 0.2	21.92 ± 1.7	9.08 ± 0.2	−0.51 ± 0.06	8	8.74 ± 0.2	44.35 ± 2.7****	8.43 ± 0.1*	−0.07 ± 0.07***	7

*^a^* The negative logarithm of the agonist concentration required to produce a half-maximal response.

*^b^* The maximal response to the ligand expressed as a percentage of the maximal cAMP production as determined using 100 μm forskolin stimulation in the presence of pertussis toxin treatment.

*^c^* The negative logarithm of the equilibrium disassociation constant for each ligand generated through use of the operational model of agonism (34).

*^d^* Log τ is the coupling efficacy parameter of each ligand.

##### Cell Line Variability in G Protein Expression

**FIGURE 5.**
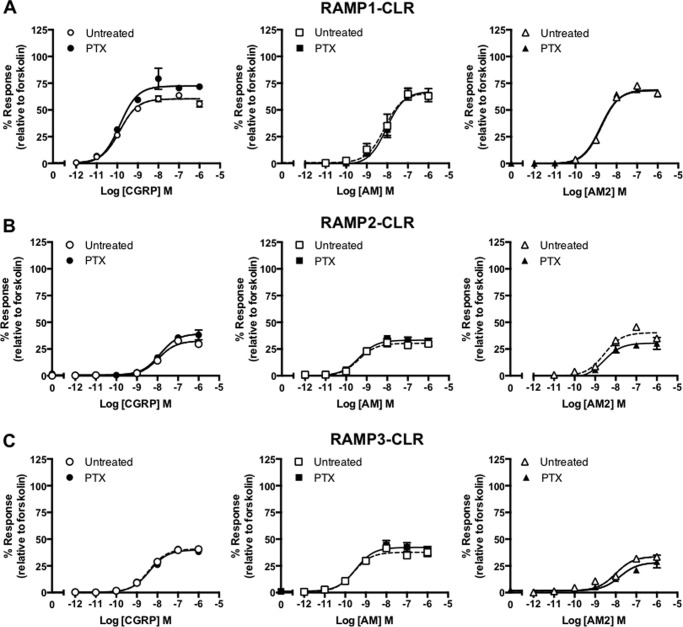
**RAMP-CLR responses appear PTX-insensitive when assayed using HEK-293S cells.** cAMP accumulation was determined in the presence (treated) and absence (untreated) of PTX from HEK-293S cells transiently transfected with CLR and RAMP1 (*n* = 5) (*A*), RAMP2 (*n* = 5) (*B*), and RAMP3 (*n* = 5) (*C*). Cells were stimulated for 30 min with CGRP, and AM2. Data are expressed as percentage of the maximal cAMP production as determined using 100 μm forskolin stimulation in the presence of PTX and are means ± S.E. of *n* individual data sets.

**FIGURE 6.**
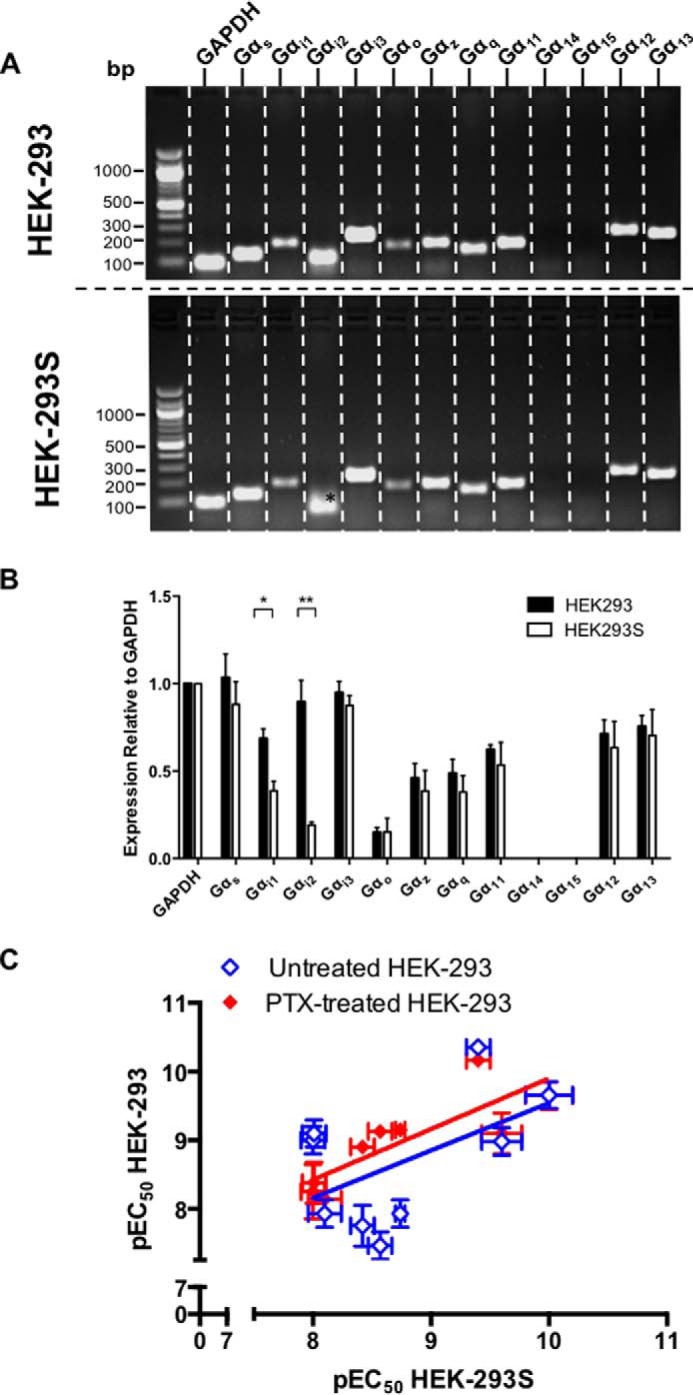
**Reduced Gα_i_ expression in HEK-293S cell lines leads to PTX insensitivity.** *A*, expression profiles of Gα genes were assessed in HEK-293 and HEK-293S cells. RNA was extracted from cells and treated with DNase 1 to remove genomic DNA contamination. Gα gene expression was examined by RT-PCR using gene-specific primers. *, indicates a lack of detectable transcript for Gα_i2_. The band shown is a nonspecific product as confirmed by DNA sequencing. *B*, semiquantitative expression (relative to GAPDH) for the Gα genes from *A* (*n* = 4). Statistical difference between HEK-293 and HEK-293S cells was determined using Student's *t* test: *, *p* < 0.05; **, *p* < 0.01. *C*, the correlation of log agonist potencies ± S.E. for CGRP, AM, and AM2 at RAMP-CLR combinations expressed in HEK-293S (Table 4) cells and HEK-293 cells either with (*red symbol*) or without (*blue symbol*) pretreatment with PTX (Table 3) was analyzed by a *scatter plot*, and Pearson's correlation coefficients (*r*) were calculated. A significant correlation was observed only between HEK-293S cells and HEK-293 cells pretreated with PTX.

The HEK-293 human cell lineage has undergone a number of modifications (35). One such lineage, HEK-293S, was adapted for growth in suspension (36). Interestingly, HEK-293S lines have also been reported to lack expression of RAMPs and therefore provide an alternative background for investigating the modulation of GPCR signal transduction (37, 38). Given that previous reports suggest that some of the effects observed with RAMPs are cell type-dependent (37, 39), we utilized HEK-293S cells as an alternative cell line. Surprisingly, and in contrast to what was observed for HEK-293 cells, HEK-293S cells pretreated with PTX and co-expressing RAMP1, RAMP2, or RAMP3 with CLR failed to demonstrate any significant change in either potency or *E*_max_ when challenged with CGRP, AM, or AM2 (Fig. 5 and Table 4; compare with Fig. 4). These results suggest that in HEK-293S cells, the RAMP-CLR combinations display little Gα_i_-mediated responses. This led us to speculate about the respective G protein content for the two cell lines. Using semiquantitative RT-PCR we assessed the expression of 12 Gα subunits (Fig. 6, *A* and *B*) in both mammalian cell lines. In the HEK-293 cells we were able to detect the expression of ten Gα subunits, with a profile similar to that documented previously for these cells (40). Transcripts were not detectable for the Gα_14_ or Gα_15_ subunits. Interestingly, in comparison with the HEK-293 cells, the HEK-293S cells displayed significantly lower expression of two Gα_i_ subunits (relative to GAPDH) but broadly similar levels of all others Gα subunits. Furthermore, there was a much better correlation between the pEC_50_ values for the ligands on HEK-293 and HEK-293S cells when the former had been pretreated with PTX, to remove the Gα_i_ component, suggesting that the differences in Gα_i_ expression between the two cell lines have functional significance (Fig. 6*C*, *r* = 0.80 (95% confidence interval, 0.27–0.96) with PTX *versus* 0.52 (95% confidence interval, −0.22 to 0.89) without PTX; *p* < 0.05). Importantly, these data demonstrate the need for caution when choosing cells for assessing G protein-mediated signaling responses.

**TABLE 4:** **Potency (pEC_50_) and maximal response (*E*_max_) for cAMP production at the CLR co-expressed with each RAMP stimulated with various agonists measured in HEK-293S cells in the presence or absence of pertussis toxin** Data are the mean ± S.E. of *n* individual data sets. No statistical difference was found between untreated and PTX-treated HEK-293S cells using Student's *t* test.

	Untreated			Treated		
	pEC_50_*^a^*	*E*_max_*^b^*	*n*	pEC_50_*^a^*	*E*_max_*^b^*	*n*
**RAMP1**						
CGRP	9.88 ± 0.1	59.98 ± 1.1	5	9.87 ± 0.1	72.92 ± 2.3	5
AM	8.13 ± 0.1	60.00 ± 3.1	5	8.03 ± 0.1	61.26 ± 2.6	5
AM2	8.74 ± 0.1	68.94 ± 1.2	5	8.78 ± 0.1	68.30 ± 1.6	5

**RAMP2**						
CGRP	8.00 ± 0.1	32.56 ± 1.0	5	7.88 ± 0.1	39.32 ± 1.7	5
AM	9.39 ± 0.1	30.34 ± 0.8	5	9.38 ± 0.1	33.28 ± 1.2	5
AM2	8.57 ± 0.1	40.30 ± 1.5	5	18.58 ± 0.16	30.52 ± 2.0	5

**RAMP3**						
CGRP	8.42 ± 0.1	40.84 ± 0.6	5	8.38 ± 0.1	39.84 ± 1.0	5
AM	9.63 ± 0.1	39.09 ± 1.2	5	9.49 ± 0.2	42.26 ± 1.6	5
AM2	8.01 ± 0.1	33.75 ± 1.5	5	7.79 ± 0.2	28.21 ± 2.4	5

*^a^* The negative logarithm of the agonist concentration required to produce a half-maximal response.

*^b^* The maximal response to the ligand expressed as a percentage of the maximal cAMP production as determined using 100 μm forskolin stimulation in the presence of PTX treatment.

##### Gα_q/11_ Coupling of CLR-based Receptors

**FIGURE 7.**
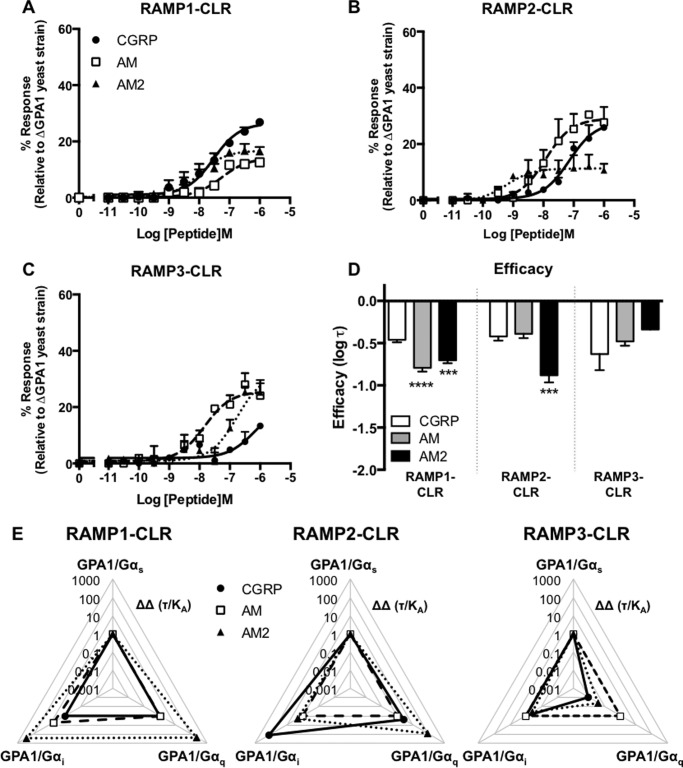
**Functional coupling of CLR co-transformed with all three RAMPs to the Gα_q_ chimera.** Dose-response curves to CGRP, AM, and AM2 were constructed in yeast strains containing the GPA1/Gα_q_ chimera and expressing CLR with RAMP1 (*n* = 7) (*A*), RAMP2 (*n* = 6) (*B*), and RAMP3 (*n* = 7) (*C*). Reporter gene activity was determined following 24-h stimulation. All data are expressed as percentage of the maximum response observed in yeast strain MMY11 and are means ± S.E. of *n* individual data sets. *D*, *Bar* chart showing the efficacy of each ligand for each RAMP-CLR combination at the Gα_q_ chimera determined via application of the operational model of receptor agonism (Ref. 34 and Table 1). Data were determined as statistically different from the cognate ligand for each receptor (***, *p* < 0.001; ****, *p* < 0.0001) using one-way ANOVA with Bonferroni's post-test. *E*, signaling bias plots were calculated as ΔΔ(τ/*K_a_*) values on a logarithmic scale for each ligand and for each chimera G protein for the three individual RAMP-CLR complexes. Determination of values requires normalization to a reference ligand (CGRP for RAMP1-CLR and AM for CLR with RAMP2 or RAMP3) and a reference pathway (in all cases, GPA1/Gα_s_).

To provide a complete investigation of the G protein coupling of the RAMP-CLR complexes, we extended our study to include the remaining nine GPA1/Gα yeast chimera-expressing strains. Coupling with the RAMP-CLR heterodimers was observed only in one additional strain that representing Gα_q_ (strain MMY89). Concentration-response curves were generated (Fig. 7, *A–C*, and Table 1), demonstrating that at RAMP1-CLR all three ligands displayed similar potencies, with CGRP being the most efficacious (log τ, Table 1) as expected for the cognate ligand at this receptor. AM2 is the most potent ligand when activating the RAMP2-CLR complex, having a reduced *E*_max_ and log τ relative to CGRP and AM (Table 1). With RAMP3-CLR, a rank order of ligand potency of AM > AM2 > CGRP was observed (Fig. 7*C* and Table 1), with all three ligands displaying broadly similar efficacies (Table 1).

##### Ligand-engendered G Protein Bias

To provide a means by which to determine the relative bias each agonist displays at each RAMP-CLR complex for the three different chimeric G proteins (in yeast), we calculated the bias factor (expressed as ΔΔ(τ/*K_a_*)) (41). For the RAMP1-CLR heterodimer, the values were calculated relative to CGRP, whereas when CLR was expressed with RAMP2 or RAMP3 the reference ligand was AM. In all cases the reference pathway used was GPA1/Gα_s_ (Fig. 7*E*). The bias plots demonstrated that at the RAMP1-CLR complex, AM2 showed a much greater bias toward signaling via GPA1/Gα_i_ and GPA1/Gα_q_ relative to CGRP, whereas AM showed a bias profile approximately equal to CGRP. With RAMP2-CLR, however, CGRP showed a much greater bias toward GPA1/Gα_i_ signaling over GPA1/Gα_s_ and GPA1/Gα_q_, whereas AM2 was more biased toward GPA1/Gα_q_. In the presence of RAMP3 all three ligands were equally biased toward GPA1/Gα_s_ and GPA1/Gα_i_, but CGRP and AM were less biased toward GPA1/Gα_q_ signaling relative to AM2.

##### Activation of RAMP-CLR Complexes Leads to Mobilization of Intracellular Ca^2+^ in Mammalian Cells

**FIGURE 8.**
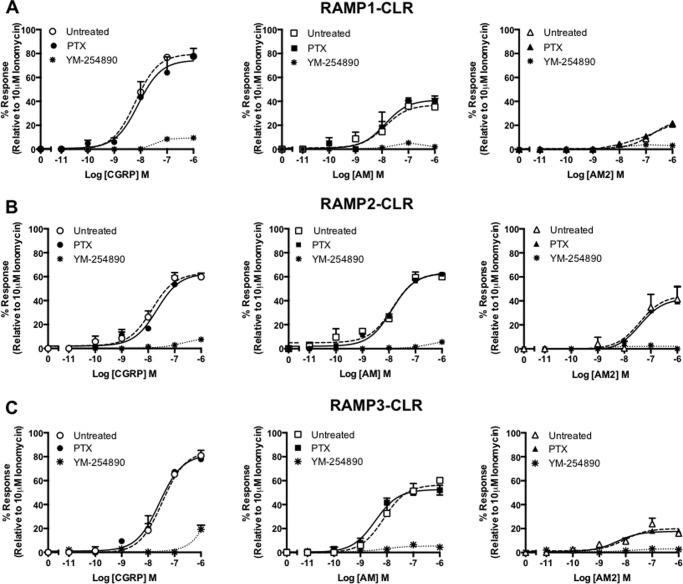
**CLR in combination with each RAMP generates receptors that mobilize (Ca^2+^)*_i_* release when expressed in HEK-293 cells.** (Ca^2+^)*_i_* mobilization was determined from HEK-293 cells transiently transfected with CLR and RAMP1 (*n* = 5) (*A*), RAMP2 (*n* = 5) (*B*), and RAMP3 (*n* = 5) (*C*). Cells were stimulated for 2 min with CGRP, AM, and AM2. Data are expressed as percentage of the maximal (Ca^2+^)*_i_* release as determined using 10 μm ionomycin. To determine the contribution made by different G proteins to the (Ca^2+^)*_i_* response, cells were preincubated with either PTX (to inhibit Gα_i_) or YM-254890 (a selective Gα_q_ inhibitor). All values are means ± S.E. of *n* individual data sets.

To confirm our findings from *S. cerevisiae*, we again utilized HEK-293 cells transiently expressing the CLR in conjunction with each RAMP and measured the release of (Ca^2+^)*_i_* upon stimulation with CGRP, AM, and AM2. Although all three ligands resulted in calcium mobilization at each RAMP-CLR complex (Fig. 8 and Table 5), these results differed slightly from that observed in *S. cerevisiae*. At both RAMP1 and RAMP2-CLR a rank order of ligand potency of CGRP = AM > AM2 was seen, whereas CGRP was the most efficacious ligand (Table 5). With RAMP3-CLR, both AM and AM2 were equipotent, with CGRP being the least potent agonist. Treatment with PTX was seen to have no effect upon the levels of calcium released in response to the three ligands, at any RAMP-CLR complex.

**TABLE 5:** **Potency (pEC_50_), affinity (p*K_a_*), and coupling efficacy (log τ) values for (Ca^2+^)*_i_* mobilization at the CLR co-expressed with each RAMP stimulated with various agonists measured in HEK-293 and HEK-293S cells** Data are the mean ± S.E. of *n* individual data sets. Statistical significance compared with the cognate ligand (*, *p* < 0.05; **, *p* < 0.01; ***, *p* < 0.001; ****, *p* < 0.0001) for each receptor heterodimer (CGRP for RAMP1-CLR and AM for CLR with either RAMP2 or RAMP3) was determined by one-way ANOVA with Dunnett's post-test.

	HEK-293					HEK-293S				
	pEC_50_*^a^*	*E*_max_*^b^*	p*K_a_**^c^*	log τ*^d^*	*n*	pEC_50_*^a^*	*E*_max_*^b^*	p*K_a_**^c^*	log τ*^d^*	*n*
**RAMP1**										
CGRP	8.19 ± 0.1	79.68 ± 0.7	7.50 ± 0.1	0.60 ± 0.05	5	8.06 ± 0.1	67.64 ± 2.0	7.57 ± 0.1	0.32 ± 0.04	5
AM	7.90 ± 0.2	37.00 ± 3.5****	7.69 ± 0.4	−0.24 ± 0.10	5	7.63 ± 0.2	38.18 ± 3.8****	7.42 ± 0.2	−0.21 ± 0.07****	5
AM2	6.76 ± 0.2***	25.05 ± 2.4****	6.64 ± 0.1**	−0.48 ± 0.06**	5	6.94 ± 0.1***	33.28 ± 1.7****	6.76 ± 0.1**	−0.30 ± 0.03****	5

**RAMP2**										
CGRP	7.86 ± 0.1	63.20 ± 3.0	7.43 ± 0.1	0.54 ± 0.10	5	7.55 ± 0.3	55.35 ± 4.5	7.21 ± 0.3	0.07 ± 0.08	5
AM	7.86 ± 0.1	63.00 ± 1.8	7.45 ± 0.2	−0.19 ± 0.06	5	7.68 ± 0.2	52.26 ± 4.5	7.39 ± 0.2	0.03 ± 0.10	5
AM2	7.41 ± 0.4	44.41 ± 7.1*	7.15 ± 0.4	−0.10 ± 0.13**	5	7.42 ± 0.4	20.17 ± 2.8****	7.33 ± 0.2	−0.65 ± 0.13***	5

**RAMP3**										
CGRP	7.47 ± 0.2*	84.39 ± 8.5*	6.66 ± 0.4*	0.74 ± 0.26	5	7.51 ± 0.2	65.3 ± 4.7	7.07 ± 0.2**	0.24 ± 0.10*	5
AM	8.12 ± 0.1	56.69 ± 6.6	7.76 ± 0.3	0.13 ± 0.13	5	8.02 ± 0.2	44.3 ± 3.2	8.56 ± 0.2	−0.11 ± 0.06	5
AM2	8.05 ± 0.3	19.99 ± 2.4*	7.95 ± 0.3*	−0.63 ± 0.08*	5	7.44 ± 0.3	20.1 ± 4.3	7.35 ± 0.3**	−0.62 ± 0.07**	5

*^a^* The negative logarithm of the agonist concentration required to produce a half-maximal response.

*^b^* The maximal response to the ligand expressed as a percentage of the maximal (Ca^2+^)*_i_* release as determined using 10 μm ionomycin stimulation.

*^c^* The negative logarithm of the equilibrium disassociation constant for each ligand generated through use of the operational model of agonism (34).

*^d^* Log τ is the coupling efficacy parameter of each ligand.

To confirm our yeast findings that the CLR can couple to Gα_q_ and thereby promote (Ca^2+^)*_i_* mobilization in mammalian cells, we utilized the known selective Gα_q/11_ inhibitor YM-254890 (42). Pretreatment with YM-254890 for 30 min prior to stimulation with AM and AM2 was sufficient to abolish all (Ca^2+^)*_i_* mobilization at all RAMP-CLR complexes. Furthermore, the response to CGRP at all three RAMP-CLR complexes was also considerably attenuated with (Ca^2+^)*_i_* release, being detected only when cells were stimulated with CGRP in the micromolar range. Similar data were obtained using HEK-293S cells (Table 5), suggesting that despite differences in Gα_i_ content, the release of (Ca^2+^)*_i_* was consistent between the two cell types. These finding suggest that all three ligands are able to initiate calcium mobilization at all three RAMP-CLR complexes in a Gαq-dependent manner in both mammalian cell lines.

##### Pathway Bias at the RAMP-CLR Complexes

**FIGURE 9.**
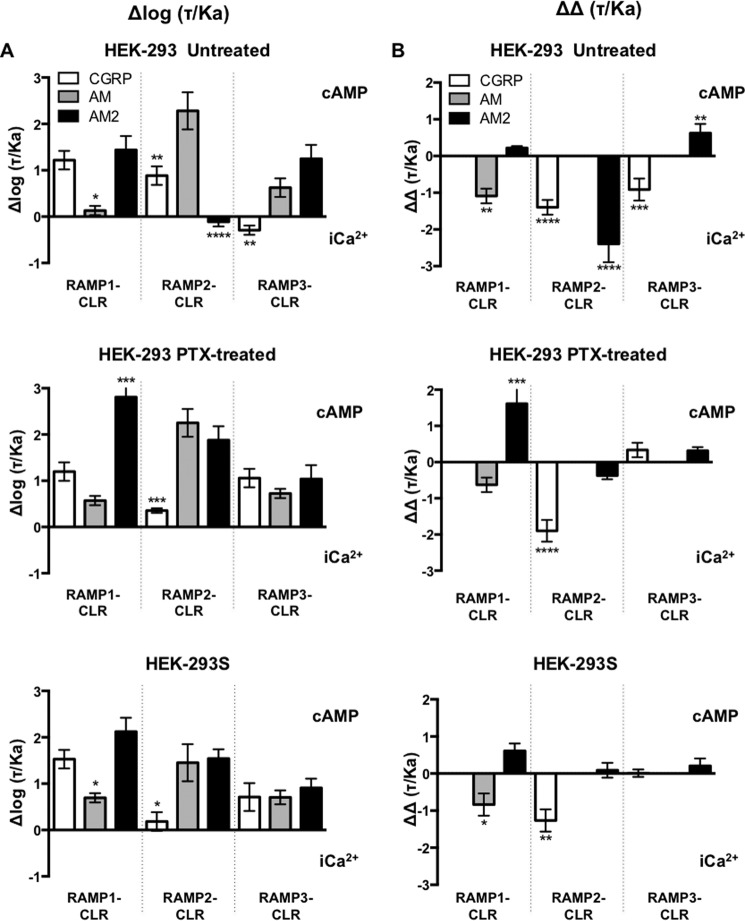
**Quantification of biased agonism at the three RAMP-CLR complexes.** *A*, normalized transduction coefficients, Δlog (τ/*K_a_*), for cAMP accumulation and (Ca^2+^)*_i_* mobilization obtained for the three RAMP-CLR complexes upon stimulated with CGRP, AM, or AM2 in untreated HEK-293 cells, HEK-293 cells treated with PTX, and HEK-293S cells. *B*, relative bias factors, ΔΔ(τ/*K_a_*), for cAMP accumulation and (Ca^2+^)*_i_* mobilization for the three individual RAMP-CLR complexes upon stimulated with CGRP, AM, or AM2 in untreated HEK-293 cells, HEK-293 cells treated with PTX, and HEK-293S cells. Determination of values requires normalization to a reference ligand (CGRP for RAMP1-CLR and AM for CLR with RAMP2 or RAMP3) and a reference pathway (in all cases cAMP accumulation). Data were determined as statistically different from the cognate ligand for each receptor (*, *p* < 0.05; **, *p* < 0.01; ***, *p* < 0.001; ****, *p* < 0.0001).

Through calculating the change in the ratio of log(τ/*K_a_*) between cAMP accumulation and the release of (Ca^2+^)*_i_*, it is possible to determine the extent of signaling bias for a ligand (Fig. 9*A*). In HEK-293 cells all ligands showed cAMP bias over (Ca^2+^)*_i_*, except for AM2 and CGRP at RAMP2-CLR and RAMP3-CLR, respectively. In contrast, in HEK-293S cells all ligands showed clear bias toward cAMP at each RAMP-CLR complex. Interestingly, treatment of HEK-293 cells with PTX generated bias profiles similar to that observed for HEK-293S cells (Fig. 9).

Further analysis of these bias factors relative to the cognate ligand at each RAMP-CLR complex (Fig. 9*B*) indicates that only AM2 displays bias toward cAMP at the RAMP1- and RAMP3-CLR complexes, whereas all other ligands display a preference to mobilize (Ca^2+^)*_i_*. Again, this is slightly different than the bias profile for HEK-293S cells. At the RAMP1-CLR complex, AM is biased toward (Ca^2+^)*_i_*, and AM2 is cAMP-biased. For RAMP2-CLR, CGRP is biased toward (Ca^2+^)*_i_* mobilization, whereas AM2 is neutral. At RAMP3-CLR all ligands are neutral and display no bias. As noted above, the inhibition of any signaling input from Gα_i_ in HEK-293 cells via PTX treatment generates a relative bias profile comparable with that seen in HEK-293S cells. Thus, we show that not only do RAMPs play a significant role in modulating signaling bias but also that cellular G protein content can drastically modulate any perceived bias.

##### Molecular Modeling of CLR and GCGR in Complex with RAMPs

**FIGURE 10.**
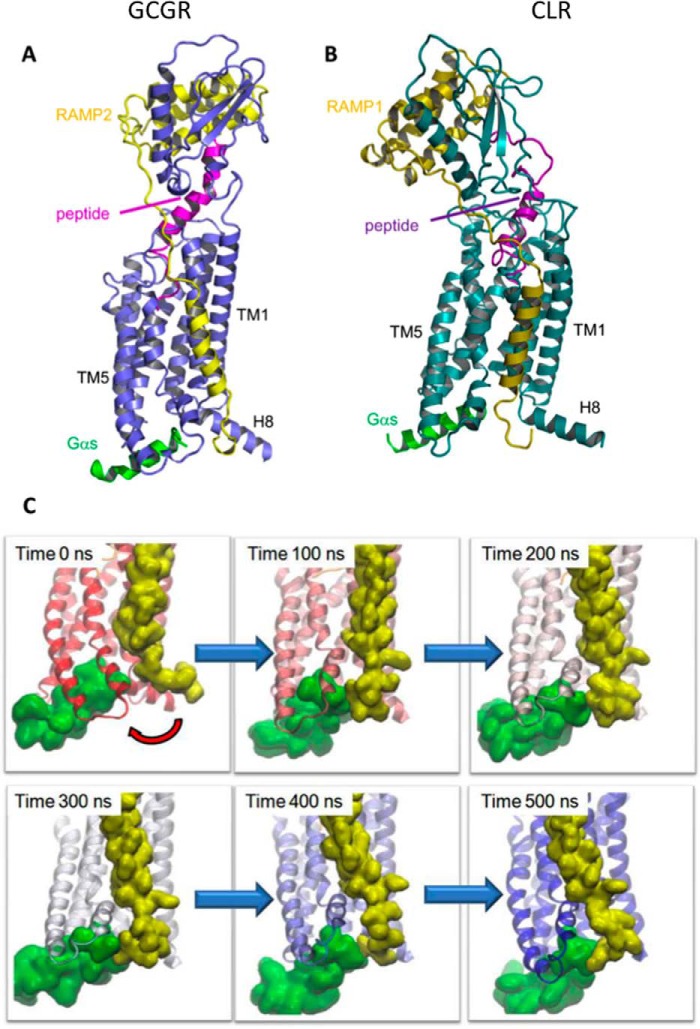
**Class B GPCR-RAMP heterodimeric models and molecular dynamics simulations.** Molecular models and dynamic simulation suggest that the C-terminal tail of RAMP1/2 (*olive*/*yellow*, when in complex with either GCGR (*A*, *blue*) or CLR (*B*, *teal*), interacts directly with the bound C-terminal of Gα_s_ (*green*) and/or helix 8. The glucagon peptide agonist is shown in *magenta*, and CGRP is shown in *purple. C*, the RAMP2 C terminus approaches the Gα_s_ (*red arrow*) during a molecular dynamics simulation of an active GCGR-RAMP2-glucagon complex. RAMP2 is shown in *yellow* and Gα_s_ in *green*, and the GCGR is colored according to time progression from *red* (0 ns) to *blue* (500 ns).

Our experimental data suggest that RAMPs may perform a critical role in modulating G protein coupling and bias. However, we do not as yet have any insight into the mechanism by which this may be achieved. To at least partially address this issue, we turned to the use of molecular modeling. We generated models of GCGR in complex with RAMP2 and CLR in complex with RAMP1. We used the GCGR system because it provides a reference system. The interaction between the peptide and the ligand is particularly well defined in the homologous GLP-1R system through reciprocal mutagenesis and photoaffinity labeling (28, 29); also we have shown that the interaction between GCGR and RAMP2 affects G protein bias (25). Models taken from the last step in the 500-ns trajectory show that in both cases, the C-terminal region of the RAMP resides in the vicinity of helix 8 (H8), the intracellular ends of TM6 and TM7, and the C-terminal region of Gα_s_ (Fig. 10, *A* and *B*). There are differences in the orientation of the extracellular domain and the precise location of the RAMP transmembrane (TM) helix due to the dynamic nature of the systems, the longer “stalk” (the region between the extracellular domain and TM1) in GCGR, and the sequence differences between the receptors and between RAMP1 and RAMP2. There are no direct interactions between the RAMPs and the peptide ligands.

Analyses of the MD trajectories show that for GCGR and CLR, the C-terminal region of the RAMP approaches the C-terminal peptide of the G protein within the first 100 ns (Fig. 10*C*). For GCGR the primary interaction is with the G protein, but there are also interactions with H8. For CLR the first part of the tail interacts with the G protein, whereas the tip of the tail interacts with H8; in both CLR and GCGR there are also interactions with the intracellular end of TM6. The interactions are driven by a combination of steric, hydrophobic, and electrostatic factors. Movies of both simulations are provided as supporting information (supplemental Movies 1 (RAMP2-GCGR-Gα_s_) and 2 (RAMP1-CLR-Gα_s_)).

**FIGURE 11.**
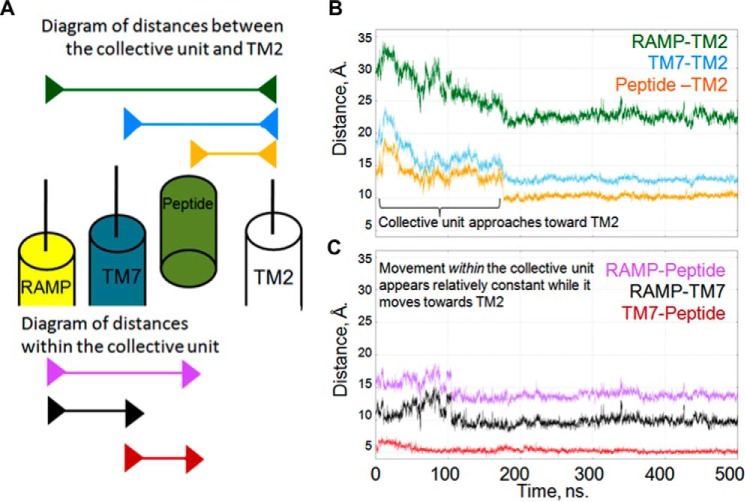
**The peptide agonist, the GPCR, and the RAMP TM helix move as a collective unit during molecular dynamics simulations.** *A*, schematic diagram of the distances between the members of the collective unit and TM2. *Top*, *arrow bars* indicate the following distances in order: RAMP2-TM2 (*green*), TM7-TM2 (*cyan*), and peptide-TM2 (*orange*). *Bottom*, *arrow bars* indicate the distances within the members of the collective unit in order: RAMP2-peptide (*purple*), RAMP-TM7 (*black*), and TM7-peptide (*red*). *B*, distances from each of the collective unit components (RAMP TM, TM7, and glucagon agonist) to TM2 (ordered as in *A*). These distances decrease in a similar manner, reflecting their concerted movement. *C*, distances between each of the collective unit components (RAMP, TM7, and glucagon agonist) (ordered as in *A*). These distances are relatively constant, reflecting their movement as a collective unit.

The extracellular end of TM7 of GCGR moves inward under the influence of RAMP2. Analysis of the distances between the extracellular end of TM2 (Cα of residue Lys-205), TM7 (Cα of residue Gly-375), the RAMP2 linker (Cα of residue Val-145), and the peptide (Cα of residue Tyr-13) shows that the RAMP TM, TM7, and the peptide move as a collective unit toward TM2 (Fig. 11), indicating a mechanism whereby the peptide ligand can influence the RAMP and vice versa even in the absence of a direct interaction.

## Discussion

The pharmacology of the CGRP family of receptors is relatively well characterized with respect to Gα_s_ coupling and the resultant accumulation of cAMP (1, 12). Gα_q_ and Gα_i_ coupling to these receptors, however, is less well characterized. Here we report the extension of the use of the *S. cerevisiae* system to investigate signaling bias in the CGRP family of receptors. These receptors are obligate heterodimers of the GPCR, namely CLR with one of three RAMPs. This dimerization adds an increased level of complexity to the system. We find that the RAMPs influence the G protein coupling in a ligand- and receptor-dependent manner, in some cases radically changing ligand selectivity.

When GPA1/Gα_i_ coupling in the yeast system was compared with coupling to GPA1/Gα_s_, markedly different responses were observed for each ligand. Most significantly, at all three receptors, the rank order of potency of the ligands was altered, either being reversed or with differences abolished. Efficacy calculations for each ligand in the presence of GPA1/Gα_i_ also revealed G protein-directed changes in the activity of each ligand. AM2 displayed a much greater efficacy at the RAMP1-CLR heterodimer than AM, and surprisingly CGRP efficacy was greatly reduced. These data indicate that the ligands display a degree of G protein bias at each receptor; this was further supported through the construction of bias plots through calculation of ΔΔ(τ/*K_a_*). The data contrast with the established potency profiles for Gα_s_-coupled receptors observed in mammalian cells and also yeast. Although Gα_s_ is recognized as the main signaling pathway activated by CLR-based receptors (15), the data illustrate that if Gα_i_ or Gα_q_ activation occurs, the conventional agonist potency ratios may lead to erroneous conclusions about the nature of the receptor. Caution should at least be taken when referring to these receptors, because it is clear that CGRP will preferentially activate the Gα_s_-coupled CGRP receptor (RAMP1-CLR), but this is not the situation when the receptor is coupled to other G proteins. Indeed, this trend is observed for all receptors in this family, with AM being the preferential ligand for both the AM_1_ (RAMP2-CLR) and AM_2_ (RAMP3-CLR) receptors coupled to Gα_s_ but not when Gα_i_ coupled. To avoid confusion we have, for the most part, described these receptors as RAMP1/2/3-CLR in this study. A further point that arises from these observations is that the reversals in potency ratios that we observed suggest that differences in the ability of the peptides to penetrate the yeast cell wall are not a contributing factor to our observations.

Our data also shed new light on the comparative efficacies of CGRP, AM, and AM2 at the three receptors for Gα_s_ coupling. Typically, they have been reported to show similar maximal responses, although there are issues with incomplete concentration-response curves (1). However, there is evidence for partial agonism of AM2 in CHO cells when RAMP2 is co-expressed with CLR (43). By its nature, the measurement of efficacy is very sensitive to the cell or tissue being studied as well as the experimental protocol. In this study, the use of the yeast assay enabled us to calculate the efficacy and potency values for each ligand-receptor combination for specific G protein subunits without the complication of pathway cross-talk. Our data revealed that all ligands have similar efficacies in cells expressing the RAMP1-CLR combination coupled to GPA1/Gα_s_. In contrast, AM has a significantly increased efficacy at the RAMP2-CLR heterodimer.

**FIGURE 12.**
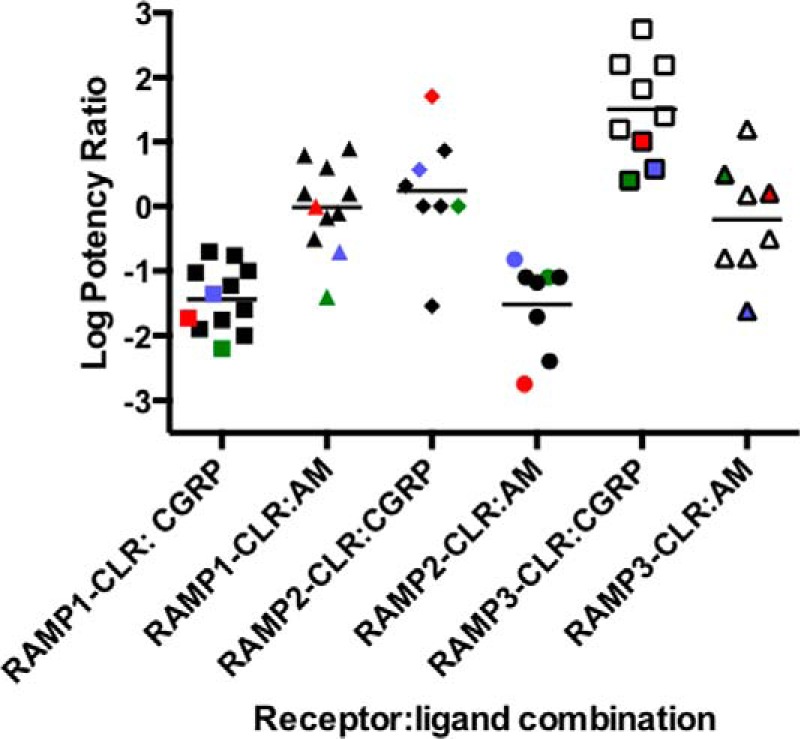
**Agonist potency ratios for CGRP, AM, and AM2 at the CLR in combination with each RAMP.** Log potency ratios (as measured by the accumulation of intracellular cAMP) are defined as log(EC_50_ AM2/EC_50_ agonist). Data are taken from Hong *et al.* (1) and others (43, 44). HEK-293 and HEK-293S cell data from the current study are shown in *red* and *blue*, respectively, and yeast Gα_s_ coupling is shown in *green*.

The relative potencies of the three peptides at the CGRP, AM_1_, and AM_2_ receptors that we observed in our current studies for Gα_s_ coupling largely agree with previous observations (1 for review, 7–9, 43, and 44) (Table 2 and Fig. 12). Importantly when each receptor was expressed in *S. cerevisiae* strains, enabling us to measure the activation of GPA1/Gα_s_, the rank potency order for the peptides fit the pattern observed in mammalian cells (Table 1 and Fig. 9), with the exception of CGRP, which displayed an unexpectedly high potency at the RAMP3-CLR heterodimer. These data indicate that, as with the GLP-1 and glucagon receptors, the yeast system is a valid model for studying G protein coupling to class B GPCRs. The comparable pharmacology of the three receptors demonstrates the value of the yeast system for assessment of the effect of complex formation by GPCRs and could be applied not only to dimerization of these receptors with RAMPs but also other modifying or downstream signaling proteins.

An important consideration is whether the Gα_i_/Gα_q_ coupling observed in yeast has any relevance to mammalian cell systems. The yeast strains express only chimeric G proteins (containing the C-terminal five amino acids of the human G protein), which are reported to be less specific when compared with equivalent G proteins expressed in mammalian cells (22). To establish the extent of Gα_i_ coupling in HEK-293 cells, we investigated cAMP production before and after PTX treatment; the greater the enhancement of cAMP production following toxin addition, the greater the extent of Gα_i_ coupling that the toxin inactivates. When compared with the coupling seen in yeast to GPA1/Gα_i_, although the correlation is not exact, there is at least a measure of agreement between the HEK-293 and yeast data, suggesting that the latter may be a guide as to what could be seen in mammalian cells given the appropriate conditions. Comparing the relative bias plots for yeast and HEK-293 cells in Figs. 7 and 9 further emphasizes this correlation; the pattern shown for the two systems is broadly similar. As the effects of RAMPs on GPCR pharmacology are known to be sensitive to the cell line background (37, 42) and significant heterogeneity in PTX sensitivity of CGRP has been reported previously (20, 45), it would perhaps be surprising if the HEK-293 cells were a perfect match to yeast. Indeed, as we have shown (Fig. 6), in terms of the expression levels of Gα_i_ subunits, two similar HEK-293 cell lines are, in fact, very different; HEK-239S cells appear to have a reduced level of Gα_i_ expression compared with HEK-293 cells. When combined with our observation of the PTX sensitivity of the CLR response in HEK-293S cell lines, it becomes apparent that we need to carefully consider the G protein content of cell lines that we utilize when investigating G protein-mediated signaling bias.

**FIGURE 13.**
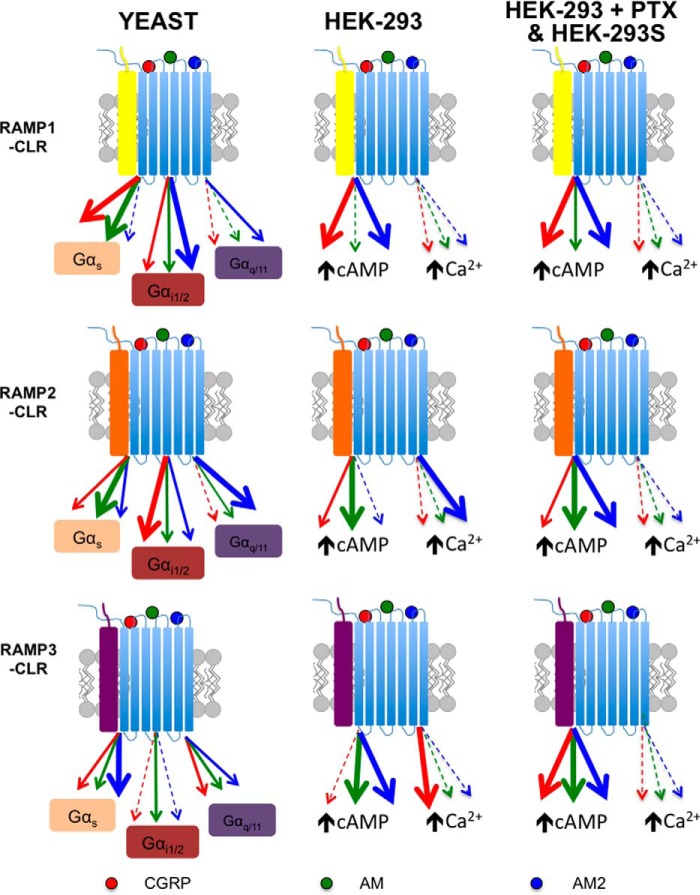
**A working model of biased agonism at the different RAMP-CLR complexes.** The individual RAMP-CLR complexes can bind the agonists CGRP (*red*), AM (*green*), and AM2 (*blue*) to activate different downstream chimeric GPA1/Gα subunits (in yeast) or promote increases in intracellular cAMP and/or mobilize release of (Ca^2+^)*_i_* (in HEK-293/HEK-293S cells). The *thickness* of the *lines* indicates the bias that each agonist displays for either the chimeric G protein or the specific downstream signaling cascade. The yeast system enabled the comparison of different individual G proteins (Gα_s_, Gα_i1/2_, and Gα_q_), whereas in mammalian cells we investigated cAMP accumulation (± PTX) and elevation of (Ca^2+^)*_i_*.

Our results have demonstrated that the CGRP family of receptors can couple to Gα_s,_ Gα_i_ and Gα_q_ subunits. Further, using the yeast system we observed a ligand-dependent G protein coupling bias with each receptor, highlighting the ability of the yeast platform to uncover potential G protein bias for other GPCRs. Importantly, this ability is, at least partially, transferred into mammalian cells and provides an excellent starting point for subsequent investigations into both the extent to which this bias occurs in native mammalian cells and the molecular basis for the phenomenon. Any examination of the physiological significance of G protein promiscuity needs to consider the cellular background in which the CLR/RAMP receptor is expressed; we observed significant differences between our three cell hosts that depend, at least partly, on the G proteins they express (Fig. 13). Indeed, it is worth highlighting that, as a direct consequence of the reduced overall Gα_i_ content in HEK-293S cells, all three ligands at the CGRP family of receptors display bias toward cAMP accumulation over (Ca^2+^)*_i_* release (Fig. 9). Coupling to Gα_i_ (or possibly Gα_o_) may be particularly relevant in neuronal and other electrically excitable cells where many (18, 19) of the effects of PTX on CGRP have been observed (reviewed in Ref. 15). In neuronal and other cells, the direct Gα_i_/Gα_o_ effects on ion channels may also be particularly significant. For example, there is the potential for a complex interplay between neuronally released CGRP and the AM or AM2 peptides released locally through cross-talk among all three CLR-based receptors, with the potential for the Gα_i_ coupling to naturally limit excitation produced via Gα_s_.

The role of Gα_q/11_ coupling in mediating responses to CGRP, AM, and AM2 has not been well investigated; the few relevant studies have examined activation of protein kinase C or release of calcium from internal stores rather than directly studying Gα_q/11_. For CGRP, a further complication is that it can also activate the amylin-1 receptor with high affinity (46), so it is not always clear that the observed effects are mediated via CLR. However, in HEK-293 cells, alveolar epithelial cells, dorsal root ganglia, and trigeminal ganglia, there is evidence for either release of intracellular calcium or activation of PKC alongside PKA activation (15). A similar pattern has been seen for AM in bovine aortic endothelial cells (47). Although evidence from PKA inhibitors such as H89 suggests that cAMP is the primary second messenger that mediates many effects of CGRP (48), there is the potential for spatial and temporal modulation of this primary signal via (Ca^2+^)*_i_*, a possibility that remains to be explored.

By utilizing molecular models of two diverse class B GPCR systems, namely the RAMP1-CLR-CGRP and RAMP2-GCGR-glucagon systems, we have gained insight into signaling bias. We believe the simulations reported here are the first molecular dynamics simulations on RAMP-GPCR heterodimers. The interaction of the RAMP TM helix with TM6/TM7 is supported both by docking experiments on CLR (27) and by studies on the secretin-GLP-1 chimeric receptor (49); this interaction remains stable throughout both 500-ns simulations of the active receptors, with the RAMP retaining a straight helix through both simulations, despite the presence of proline(s). The interaction is primarily with TM7 and the N-terminal end of TM6. This provides some evidence that GCGR and CLR may interact with RAMP in a similar way. Despite the persistence and stability of the TM interactions, the C terminus is quite flexible, sampling a wide region of space in both simulations. RAMP2 interacted primarily with the C terminus of Gα_s_, whereas RAMP1 interacted primarily with H8 but also made contact with TM6 and most importantly Gα_s_. These simulations therefore indicate that the RAMP could affect the bias shown in G protein coupling by CLR either by direct interaction and/or allosterically by altering the orientation of TM6 and TM7 or H8. These simulations were carried out on a model of the active receptor in complex with a C-terminal fragment of Gα_s_ (Arg-374 to Leu-394). The C-terminal helix of Gα_s_ sits above the face of the G protein. Models of RAMP2-GCGR in complex with the G protein heterotrimer indicated that the RAMP could also interact directly with residues around Gly-353 of Gα_s_ (results not shown).

In addition, the allosteric effects of the RAMP linker may alter the extracellular face of the receptor (as seen in CLR with RAMP2 and RAMP3 (27)), and these effects could be transmitted to the intracellular end of the helix. In our simulations, we see some evidence for the top of TM7 moving in toward the TM bundle under pressure of the RAMP (Fig. 11) as part of a collective unit comprising TM7, the peptide, and the RAMP TM. This concerted movement provides a possible mechanism whereby the influence of the ligand can be conveyed to the RAMP and thereby affect the bias via interactions of the C terminus of the RAMP. The inward movement of the extracellular end of TM7 has been linked explicitly to activation (50), but movement of TM6 and/or H8 under the influence of the RAMP may also affect bias and activation. Thus we suggest that RAMPs have the potential to interact allosterically with not only the GPCR but also the bound G protein. This leads to the possibility that, upon ligand binding, the RAMPs contribute to the G protein bias. To confirm this likelihood, we aim to extend this project to investigate all ligand-RAMP-CLR-G protein complexes and further elucidate the role that RAMPs play in modulating G protein coupling and bias at the CGRP family of receptors.

Finally, we suggest that this study has broader implications. Our results shown here are similar to those described for the GCGR (25) in that RAMPs alter the ability of peptides to stimulate different G proteins. However, as we have shown, significant pharmacological differences can be observed in differing recombinant cell lines and expression systems. These differences can be explained through several factors; these systems rely firstly upon overexpression of the receptor and chaperone proteins under study and secondly upon the cellular content of further downstream signaling proteins such as G proteins. It is therefore important that findings in systems such as those explained here be further validated. This would be best achieved in cell lines endogenously expressing the GPCR and RAMP of interest. This is thus something that we aim to undertake as a follow-up to the work presented here for CLR-RAMP complexes. It is clear that there is a complex interplay among the ligand, the RAMP, and the CLR that alters G protein activation for these receptors. Further, our data presented here add to the growing wealth of literature suggesting that many ligands for class B GPCRs display either a Gα_s_ or Gα_i_ signaling preference. To date, this ligand-engendered bias has been observed for receptors binding corticotropin-releasing factor, urocortin 1, GLP-1, and glucagon (24, 25, 51). In the current study, the yeast growth assay system was able to provide a valuable indication of the potential of the CGRP family of receptors to couple to either Gα_s_ or Gα_i_ when stimulated by CGRP, AM, or AM2, allowing us to uncover novel G protein signaling preferences for each ligand. We therefore conclude that this system is a good platform from which to explore the effect of RAMP dimerization to other members of the class B GPCRs.

## Experimental Procedures

### 

#### 

##### Materials

Human (h) αCGRP, hAM, and hAM2 (1–47) were purchased from Bachem (Bubendorf, Switzerland) and made to 1 mm stocks in water containing 1% BSA. Yeast nitrogen base and yeast extract were purchased from Difco (Franklin Lakes, NJ). Fluorescein-di-β-d-glucopyranoside was purchased from Invitrogen. Forskolin was from Tocris Bioscience (Wiltshire, UK), and YM-254890 was supplied by Alpha Laboratories (Hampshire, UK). Both the ALPHAScreen and LANCE® cAMP detection assay kits and all reagents were from PerkinElmer Life Sciences.

##### Expression Constructs

To enable expression of the human CLR, we used either a previously described (25) Myc-tagged cDNA construct provided by Dr. Michel Bouvier (University of Montreal, Canada) or a human CLR with an N-terminal HA epitope tag. All human FLAG-tagged RAMPs were used as described previously (37).

##### Yeast Strain Construction and Assay

General yeast procedures were performed as described previously (22, 24). The human CLR was introduced into yeast cells under the control of the *PGK* promoter using a plasmid containing *ura3* (pDT-PGK). The three human RAMPs were introduced into yeast under the control of the *GAPDH* promoter using plasmids containing *leu2* (p425-GPD) (25). *S. cerevisiae* dual reporter strains expressing chimeras of yeast GPA1(1–467) (GPA1/Gα) with the five C-terminal amino acids of 11 human G proteins representing Gα_s_, Gα_16,_ Gα_q,_ Gα_o,_ Gα_i1/2,_ Gα_i3,_ Gα_z,_ Gα_12,_ Gα_13,_ and Gα_14_ (MMY84–MMY93) were used in this study (52). The human CLR and RAMPs were transformed into yeast cells (at a ratio of 1:1 to enable equal expression) using the lithium acetate/single-stranded DNA/polyethylene glycol method as described previously (53). Positive transformants were selected and maintained on synthetic dropout (SD) medium lacking both uracil and leucine (SD-Ura-Leu). Receptor signaling was measured using the yeast growth assay as described previously (24). Cell growth was initially performed in SD-Ura-Leu medium at 30 °C to select cells expressing only both plasmids. Cells were then cultured to remove basal activity in SD-Ura-Leu-His medium overnight at 30 °C and assayed using medium supplemented with fluorescein-di-β-d-glucopyranoside. A fluorescein signal was detected as an increase in fluorescence (excitation wavelength = 485 nm, emission wavelength = 535 nm) as a measure of growth. Different concentrations of ligand (0.01 nm–100 μm) were assayed using 96-well plates, and fluorescence was detected using a Tecan Infinite M200 microplate reader (Tecan Ultra Evolution, Reading, UK) or a Mithras LB 940 microplate reader (Berthold Technologies, Harpenden, UK) for 20 h. Positive isolates were selected for their ability to grow above basal level in SD-Ura-Leu-His medium, when stimulated with 10 μm CGRP or AM as appropriate for the RAMP-CLR complex being studied. Chimeric strains were deemed not to functionally couple when *n* > 16 isolates had been assayed and none showed growth above basal levels. In this study functional couplings were only observed for MMY84, MMY86, and MMY88 representing Gα_s_, Gα_i1/2_, and Gα_q_, respectively.

##### Mammalian Cell Culture and Transfection

HEK-293 cells, provided by Dr. Jügen Müller (University of Aston), were cultured in DMEM supplemented with 10% heat-inactivated FBS and kept at 37 °C in a humidified 95% air, 5% CO_2_ incubator. HEK-293S cells (a gift from AstraZeneca) were cultured in DMEM supplemented with 8% heat-inactivated FBS and kept at 37 °C in a humidified 95% air, 5% CO_2_ incubator. HEK-293 cells were transfected with FuGENE 6 (Roche Applied Science) in accordance with the manufacturer's instructions using a 1:3 (w:v) DNA:FuGENE ratio and a 1:1 ratio of RAMP to CLR. HEK-293S cells were seeded into 96-well poly-d-lysine-coated plates at a density of 15,000 cells/well (determined using a Countess^TM^ cell counter, Invitrogen) 1 day prior to transfection. HEK-293S cells were transiently transfected as described previously (38) using a 1:1 ratio of RAMP to CLR. The transfected cell lines were grown for 24–48 h prior to assaying. Where appropriate, PTX (200 ng/ml) was added to ADP-ribosylate Gα_i_ for 16 h prior to assaying, thereby uncoupling receptor-mediated Gα_i_-dependent inhibition of cAMP production.

##### cAMP Accumulation Assays

The transfected HEK-293 cells were washed in PBS, resuspended in stimulation buffer (PBS containing 0.1% BSA and 0.5 mm IBMX), and seeded at 2000 cells/well in 384-well white Optiplates. Ligands were added in the range of 1 pm to 1 mm, and cAMP accumulation was measured after 30 min of stimulation using a LANCE® cAMP detection kit (PerkinElmer Life Sciences). We had found previously that a 30-min stimulation was the optimum time for assaying cAMP accumulation for family B GPCRs (24, 25). Plates were read using a Mithras LB 940 multimode microplate reader (Berthold Technologies). HEK-293S cells were assayed for cAMP accumulation as described elsewhere (54). Values were converted to concentration using a cAMP standard curve performed in parallel.

##### Calcium Mobilization Assays

Transfected HEK-293 cells were grown to confluence in black, clear bottomed, 96-well plates. On the day of assay cells were washed with calcium-free Hanks' balanced salt solution and incubated for 1 h at room temperature in the presence of 10 μm Fluo-4/AM (Invitrogen) containing 2.5 mm probenecid. Cells were then washed followed by the addition of 100 μl of Ca^2+^-free Hanks' balanced salt solution. Ligands were added robotically using a Mithras LB 940 multimode microplate reader in the range of 10 pm to 1 μm, and fluorescence was determined immediately post-injection with an excitation wavelength set to 485 nm and an emission wavelength set to 535 nm. Recordings were obtained every 0.5 s for 120 s. Peak magnitude was calculated using five-point smoothing followed by correction against background fluorescence. The peak was used to generate concentration-response curves and normalized relative to 10 μm ionomycin. To determine the role played by Gα_q/11_ in (Ca^2+^)*_i_* mobilization, cells were pretreated (for 30 min) with 100 nm YM-254890, which inhibits Gα_q/11_ signaling (42).

##### RT-PCR

RNA was extracted from HEK-293 and HEK-293S cells using a RNAqueous-4PCR kit (ThermoFisher Scientific) as per the manufacturer's protocol. All RNA samples were treated with DNase I to remove contaminating genomic DNA. Reverse transcription was performed using a QuantiTect reverse transcription kit (Qiagen, Manchester, UK). The PCR amplification was performed as described previously (55) using gene-specific primers to human Gα subunits: Gα_s_, forward (5′-CGACGACACTCCCGTCAAC-3′) and reverse (5′-CCCGGAGAGGGTACTTTTCCT-3′) (PrimerBank ID, 3297877a1 (56)); Gαi_1_, forward (5′-TTAGGGCTATGGGGAGGTTGA-3′) and reverse (5′-GGTACTCTCGGGATCTGTTGAAA-3′) (PrimerBank ID, 156071490c1 (56)); Gαi_2_, forward (5′-TACCGGGCGGTTGTCTACA-3′) and reverse (5′-GGGTCGGCAAAGTCGATCTG-3′) (PrimerBank ID, 261878574c1 (56)); Gαi_3_, forward (5′-ATCGACCGCAACTTACGGG-3′) and reverse (5′-AGTCAATCTTTAGCCGTCCCA-3′) (PrimerBank ID, 169646784c1 (56)); Gαq, forward (5′-TGGGTCAGGATACTCTGATGAAG-3′) and reverse (5′-TGTGCATGAGCCTTATTGTGC-3′) (PrimerBank ID, 312176363c1 (56)); Gα_11_, forward (5′-GGCTTCACCAAGCTCGTCTAC-3′) and reverse (5′-CACTGACGTACTGATGCTCG-3′) (PrimerBank ID, 115511048c1) (56)); Gα_z_, forward (5′-GGTCCCGGAGAATTGACCG-3′) and reverse (5′-ATGAGGGGCTTGTACTCCTTG-3′) (PrimerBank ID, 45580725c1) (56)); Gα_0_, forward (5′-GGAGCAAGGCGATTGAGAAAA-3′) and reverse (5′-GGCTTGTACTGTTTCACGTCT-3′) (PrimerBank ID, 162461737c1 (56)); Gα_12_, forward (5′-CCGCGAGTTCGACCAGAAG-3′) and reverse (5′-TGATGCCAGAATCCCTCCAGA-3′) (PrimerBank ID, 42476110c1) (56)); Gα_13_, forward (5′-CAGCAACGCAAGTCCAAGGA-3′) and reverse(5′-CCAGCACCCTCATACCTTTGA-3′) (PrimerBank ID, 215820623c1) (56)); Gα_14_, forward (5′-GAGCGATGGACACGCTAAGG-3′) and reverse (5′-TCCTGTCGTAACACTCCTGGA-3′) (PrimerBank ID, 222418795c1 (56)); Gα_15_, forward (5′-CCAGGACCCCTATAAAGTGACC-3′) and reverse (5′-GCTGAATCGAGCAGGTGGAAT-3′) (PrimerBank ID, 156104882c1 (56)); and *GAPDH*, forward (5′-AATGGGCAGCCGTTAGGAAA-3′) and reverse (5′-GCGCCCAATACGACCAAATC-3′). All products were resolved on a 2% agarose gel and imaged using a G:Box iChemi gel documentation system utilizing GeneTools analysis software (Syngene, Cambridge, UK), and densitometry was performed using GeneTools.

##### Molecular Modeling

Models of the GCGR in complex with RAMP2 and CLR in complex with RAMP1 were based on the previously reported models of GLP-1R in complex with GLP-1 and CLR in complex with RAMP2/3, respectively (27–29). These models were built using MODELLER 9.16 (57) from the GCGR and CRFR x-ray structures of the TM domain (58, 59), the x-ray structures of the extracellular domain (60, 61), and NMR structures of closely related peptides (62, 63). The helical region of the CGRP peptide was structurally aligned to the corresponding region in GLP-1 based on the sequence alignment (27) because the position of the GLP1 helix within GLP-1R is well defined by experimentation; the initial models are available as supporting information. The RAMP-GPCR complexes were placed in a hydrated POPC membrane using CHARMM GUI (64) to generate a system containing 20,482 and 28,013 TIP3P water molecules (65), as well as 183 and 243 lipid molecules for the RAMP2-GCGR and RAMP1-CLR heterodimers, respectively. The histidine protonation was determined using the PDB2PQR server (66). The AMBERSP99 force field parameters for the protein (67), and the lipid14 force field parameters for POPC (68, 69) were added using AmberTools (70). Molecular dynamics simulations were run for 500 ns at 298 K using ACEMD (71).

##### Data Analysis

Data analysis for cAMP assays was performed in GraphPad Prism 6.0f (San Diego, CA). Data were fitted to obtain concentration-response curves using either the three-parameter logistic equation (for pEC_50_ values) or the operational model for partial agonism (34) to obtain values of efficacy (log τ) and the equilibrium dissociation constant (log *K_a_*). These values were then used to quantify signaling bias as change in log (τ/*K_a_*) relative to the natural cognate ligand for the respective receptor (41). We denoted these as CGRP for CLR with RAMP1 and AM for CLR with either RAMP2 or RAMP3. Statistical differences were analyzed using one-way ANOVA or Student's *t* test as appropriate with post hoc Bonferroni's or Dunnett's multiple comparisons, and *p* < 0.05 was considered significant. Correlations between pEC_50_ values for cAMP assays of HEK-293 and HEK-293S cells were assessed by scatter plot and Pearson's correlation coefficient (*r*). For RT-PCR, normalization to the internal standard GAPDH was performed to reduce variance and enable a comparison between different cell lines. To quantitate the ligand-dependent response in the yeast system, a strain lacking GPA1 (MMY11), grown in rich medium, was used as a standard (72). As GPA1 is not present in this strain, the Gβγ subunits are unregulated and free to signal, allowing us to determine the maximal response of our system. *E*_max_ values are reported as a percentage of this maximum response, and statistical analysis was performed on these data. For the mammalian cell-based assays, data analysis was carried out as for the yeast curves. To account for the day-to-day variation experienced from transient transfections, we used the maximal level of cAMP accumulation from cells in response to 100 μm forskolin stimulation as our reference and 10 μm ionomycin for (Ca^2+^)*_i_* assays. *E*_max_ values from these curves are reported as a percentage of these controls, and all statistical analysis has been performed on these data. Where appropriate the operational model for partial agonism (34) was used to obtain efficacy (log τ) and equilibrium disassociation constant (log *K_a_*) values. In both cases, this normalization removes the variation due to differences in transfection or transformation but retains the variance for control values. The means of individual experiments were combined to generate the curves shown.

## Author Contributions

C. W., H. A. W., and G. L. conceived and designed the research. C. W., A. S., and R. H. performed the yeast experiments, and I. W. and H. A. W. performed the mammalian assays. M. H. performed the RT-PCR. C. A. R., J. C. M., and D. A. W. carried out the computational chemistry, and S. J. D. provided yeast strains. C. W., D. R.P., H. A. W., J. C. M., C. A. R., and G. L. analyzed the data. C. W., D. R. P., H. A. W., J. C. M., C. A. R., and G. L. wrote the manuscript, and S. J. D. revised and edited the manuscript.

## Supplementary Material

Supplemental Data

## Footnotes

3 The abbreviations used are:

CGRP: calcitonin gene-related peptide
AM: adrenomedullin
GPCR: G protein-coupled receptor
CLR: calcitonin receptor-like receptor
RAMP: receptor activity-modifying protein
POPC: 1-palmitoyl-2-oleoyl-*sn*-glycero-3-phosphocholine
PTX: pertussis toxin
GLP: glucagon-like peptide
GCGR: glucagon receptor
TM: transmembrane
ANOVA: analysis of variance.
