# Supplementary material for: Receptor Activity-modifying Protein-directed G Protein Signaling Specificity for the Calcitonin Gene-related Peptide Family of Receptors
Source: J Biol Chem. 2016 Aug 26;291(42):21925–44. doi: 10.1074/jbc.M116.751362 (PMC5063977; doi:10.1074/jbc.M116.751362)
Supplement: Supplemental Data [file supp_291_42_21925__index.html]

Receptor activity modifying protein-directed G protein signaling specificity for the calcitonin gene-related peptide family of receptors — Receptor Activity-modifying Protein-directed G Protein Signaling Specificity for the Calcitonin Gene-related Peptide Family of Receptors — G Protein Bias in CLR-based Receptors — Supplemental Data 

# Receptor Activity-modifying Protein-directed G Protein Signaling Specificity for the Calcitonin Gene-related Peptide Family of Receptors

## Supplemental Data

- Supplemental Figures Legend (.pdf, 55 KB)
