# Supplementary material for: Receptor Activity-modifying Protein-directed G Protein Signaling Specificity for the Calcitonin Gene-related Peptide Family of Receptors
Source: J Biol Chem. 2016 Aug 26;291(42):21925–44. doi: 10.1074/jbc.M116.751362 (PMC5063977; doi:10.1074/jbc.M116.751362)
Supplement: Supplemental Data [file 10.1074_M116.751362_jbc.M116.751362-1.pdf]

Supplementary Movie 1.
The RAMP2 C-terminus (yellow surface, right hand side) approaches toward Gα_s (green surface, left hand side) during a molecular dynamics simulations of an active RAMP2-GCGR-glucagon-Gα_s complex. The GCGR (ribbon representation) is coloured according to time progression, from red (0 ns) to blue (500 ns).

Supplementary Movie 2.
The RAMP1 C-terminus (yellow surface, right hand side) approaches toward Gα_s (blue surface, left hand side) during a 500 ns molecular dynamics simulations of an active RAMP1-CLR-CGRP-Gα_s complex. CLR (ribbon representation) is coloured green. Part of the RAMP C-terminus also contacts H8 of CLR.
